# The Aqueous Extract of Eucommia Leaves Promotes Proliferation, Differentiation, and Mineralization of Osteoblast-Like MC3T3-E1 Cells

**DOI:** 10.1155/2021/3641317

**Published:** 2021-06-19

**Authors:** Mengqi Guan, Daian Pan, Mei Zhang, Xiangyang Leng, Baojin Yao

**Affiliations:** ^1^College of Traditional Chinese Medicine, Changchun University of Chinese Medicine, Changchun, Jilin 130117, China; ^2^Jilin Ginseng Academy, Changchun University of Chinese Medicine, Changchun, Jilin 130117, China; ^3^Innovation Practice Center, Changchun University of Chinese Medicine, Changchun, Jilin 130117, China

## Abstract

Eucommia leaves are dry leaves of *Eucommia ulmoides* which have long been considered as a functional health food for the treatment of hypertension, hypercholesterolemia, fatty liver, and osteoporosis. With the recent development of Chinese medicine, Eucommia leaves are widely used for tonifying the kidneys and strengthening bone. However, the specific molecular mechanism of Eucommia leaves for strengthening bone remains largely unknown. Osteoblasts are the main functional cells of bone formation; thus, it is essential to study the effect of Eucommia leaves on osteoblasts to better understand their mechanism of action. In the present study, we prepared an aqueous extract of Eucommia leaves (ELAE) and determined its content by high-performance liquid chromatography (HPLC). The effects of ELAE on MC3T3-E1 cells were investigated by CCK-8 assay, alkaline phosphatase (ALP), and Alizarin red S staining assays, combined with RNA sequencing (RNA-seq) and qRT-PCR validation. We demonstrated that ELAE had a significant promoting effect on the proliferation of MC3T3-E1 cells and significantly enhanced extracellular matrix synthesis and mineralization, which were achieved by regulating various functional genes and related signaling pathways. ELAE significantly increased the expression level of genes promoting cell proliferation, such as Rpl10a, Adnp, Pex1, Inpp4a, Frat2, and Pcdhga1, and reduced the expression level of genes inhibiting cell proliferation, such as Npm1, Eif3e, Cbx3, Psmc6, Fgf7, Fxr1, Ddx3x, Mbnl1, and Cdc27. In addition, ELAE increased the expression level of gene markers in osteoblasts, such as Col5a2, Ubap2l, Dkk3, Foxm1, Col16a1, Col12a1, Usp7, Col4a6, Runx2, Sox4, and Bmp4. Taken together, our results suggest that ELAE could promote osteoblast proliferation, differentiation, and mineralization and prevent osteoblast apoptosis. These findings not only increase our understanding of ELAE on the regulation of bone development but also provide a possible strategy to further study the prevention and treatment of osteogenic related diseases by ELAE.

## 1. Introduction

*Eucommia ulmoides* is also known as duzhong (in Chinese), tusipi, gutta-percha tree, sizhong, and sixian. *Eucommia ulmoides* is widely distributed in China [1], and its leaves have been widely used in China and abroad due to their high medicinal and commercial value. The tea made from Eucommia leaves is considered healthy food with high medicinal value [2]. Studies have shown that Eucommia leaves have rich pharmacological activity, including antioxidant effects [1], antibiosis, anti-inflammatory effects [3], blood pressure lowering effects [4, 5], and bone growth [6].

MC3T3-E1 is an osteoblast precursor cell line derived from the mouse parietal region; it is often used as an experimental model to evaluate osteogenic differentiation and proliferation ability [7]. During differentiation, osteoblasts can express extracellular matrix proteins, such as alkaline phosphatase (ALP), osteocalcin, and other bone matrix proteins [8].

The discovery of natural drugs is important for addressing global health challenges and achieving sustainable health development goals [9]. There are many ways to develop natural drugs, of which RNA sequencing (RNA-seq) is one of the important ones. RNA-seq has been applied to drug screening and biomarker detection of drugs because of its high throughput, rapid detection speed, and simple analysis methods [10]. In addition, RNA-seq has also been widely used in bone biology research to address problems of bone development and bone diseases [11].

In this study, we prepared the aqueous extract from Eucommia leaves with the optimal yield as the standard. The main components of the aqueous extract were detected by high-performance liquid chromatography (HPLC). The Cell Counting kit-8 (CCK-8) experiment demonstrated that Eucommia leaves had cell proliferation effects on osteoblasts. Alizarin red staining and alkaline phosphatase staining also demonstrated the effect of Eucommia leaves on osteoblasts. Transcriptome analysis of the effect of Eucommia leaves on osteoblast MC3T3-E1 was conducted using advanced RNA-seq technology, and the differentially expressed genes were screened by bioinformatics analysis. These results provide ideas for revealing the effects of the aqueous extract of Eucommia leaves on osteoblasts and osteoblast-related bone diseases.

## 2. Materials and Methods

### 2.1. Collection and Authentication of the Plant

The Eucommia leaves of *Eucommia ulmoides* (Figure S1) were collected from Zhangjiajie city, Hunan province of People's Republic of China, and identified at the Changchun University of Chinese Medicine by Professor Jinglei Xiao.

### 2.2. ELAE Preparation and HPLC Analysis

The aqueous extract was prepared by boiling under optimum conditions, and the filtrate was freeze-dried with a Heto PowerDry LL3000 freeze dryer (Thermo, USA) and stored at −80°C. The components and content of Eucommia leaf extracts were determined by HPLC. The standard chemicals (aucubin, geniposidic acid, carnosine, catechin, isoquercitrin, chlorogenic acid, and astragaloside) were purchased from Shanghai Yuanye Biotechnology Co., Ltd (Yuanye, China). HPLC analysis of ELAE was performed using a 2695 liquid chromatographic system equipped with an inverted C18 column (Waters, USA). The mobile phase was a gradient elution system composed of water containing acetonitrile (A)-0.1% phosphoric acid solution (B), with a flow rate of 1 ml/min, and a column temperature of 35°C. Acetonitrile (A)-0.1% phosphoric acid solution (B) was eluted in a gradient manner (0–7 min, 5% A; 7–9 min, 5% A ⟶ 8% A; 9–28 min, 8% A; 28–30 min, 8% A ⟶ 20% A; 30–42 min, 20% A; 42–43 min, 20% A ⟶ 50% A; 43–63 min, 50% A; 63–64 min, 50% A ⟶ 5% A). A photodiode array (PDA) detector was set at 254 nm, and the online UV spectrum was recorded within the range of 200–300 nm.

### 2.3. Cell Proliferation Assay of MC3T3-E1 Cells

Cell Counting kit-8 (CCK-8, Sigma, USA) and colony formation assay were used to analyze proliferation. MC3T3-E1 osteoblasts were cultured by subculture with the passage number less than 10, and the cell suspension was obtained. Then, 3 × 10^3^ cells/ml (100 *μ*L per well) were inoculated in 96-well cell culture plates and incubated at 37°C in a 5% CO_2_ incubator (Thermo, USA) for 24 h to reach the population doubling time (PDT). MC3T3-E1 cells were treated with ELAE at different concentrations (0, 0.05, 0.1, 0.15, 0.2, 0.25, 0.3, and 0.35 mg/mL dissolved in culture medium (100 *μ*L per well) and cultured in an incubator for 24 h. Subsequently, 10 *μ*L of CCK-8 reagent was added to each well in the dark and incubated for 60 min. We evaluated cell viability by measuring absorbance at 450 nm using a microplate reader (Tecan, Switzerland).

### 2.4. Alkaline Phosphatase Assay

MC3T3-E1 cells were treated for 24 h in 6-well plates with ELAE. Cells were fixed at room temperature in 95% ethanol for 20 min, rinsed with PBS, and stained with BCIP/NBT alkaline phosphatase (ALP) staining kit (Beyotime, China) in accordance with the manufacturer's instructions. Images were taken with an optical microscope (Olympus, Japan) accompanied by a digital camera. To determine the enzyme activity of alkaline phosphatase, MC3T3-E1 cells were treated as described above, and the proteins were extracted. The alkaline phosphatase enzyme activity was measured using an alkaline phosphatase test kit (Beyotime, China) in line with the manufacturer's instructions. The absorbance of alkaline phosphatase activity was measured at 405 nm using a microplate reader (Tecan, Switzerland).

### 2.5. Alizarin Red Staining Assay

The cells were either treated by ELAE at an optimum concentration based on the result of the cell proliferation assay or treated with a plain culture medium (Thermo, USA) and incubated for 24 h. All culture media were removed, and cells were washed with phosphate-buffered saline buffer (Thermo, USA). The cells were fixed by incubating with 4% paraformaldehyde for 15 min at room temperature. After washing with Millipore purified water three times, the fixed cells were stained with Alizarin red S staining solution (Solarbio, China) for 30 min at room temperature and washed with purified water three times. Images were taken with an optical microscope (Olympus, Japan) accompanied by a digital camera. Quantitative analysis was carried out after dissolving the stained cells with cetylpyridinium chloride monohydrate reagent (Solarbio, China) for 1 h at room temperature, using a plate reader (Life science, USA) at an optical density (OD) of 562 nm.

### 2.6. RNA Purification and Illumina Sequencing

MC3T3-E1 cells were inoculated with 1 × 10^6^ cells/well in 6-well cell culture plates and incubated in an incubator (37°C, 5% CO_2_) for 12 h. Next, either ELAE at an optimum concentration based on the result of the cell proliferation assay or plain culture medium (Thermo, USA) was added to the corresponding 6-well cell culture plate and incubated for 24 h. All media were removed, and the cells were gently rinsed with a precooled phosphate-buffered saline buffer (Thermo, USA). TRIzol (Invitrogen, USA) was used to separate total RNA. The integrity of RNA was assessed using an Agilent 2100 bioanalyzer (Agilent Technologies, USA). According to the manufacturer's proposal, a paired-end messenger RNA libraries were generated using the Tru-Seq Stranded mRNA kit (Illumina, USA). High-throughput sequencing was performed on the Illumina Hi-Seq 2500 platform (Illumina, USA).

### 2.7. RNA-Seq Data Analysis

After RNA-seq, the image data output was transformed via base calling into raw data in FASTQ format. The raw reads were trimmed to remove the low-quality reads and adapter sequences and to generate clean reads. The data sets were uploaded to the NCBI Sequence Read Archive (SRA) database (accession number: PRJNA690876). Subsequently, the reads were aligned with the mouse (*Mus musculus*) reference genome using the HISAT program [12]. Gene expression levels were measured using the FPKM algorithm [13]. The BLAST program was used to perform annotations against the nonredundant (NR) and Swiss-Prot protein databases. Differentially expressed genes were analyzed by the DEG-seq program [14]. Genes with a log_2_ fold change ≥1 or ≤−1 and with a *p* value ≤ 0.001 were considered as differentially expressed genes (DEGs).

### 2.8. Quantitative Real-Time PCR Verification

Quantitative real-time PCR (qRT-PCR) was used to verify the RNA levels of the selected genes. cDNA was generated from a total RNA using iScript cDNA synthesis kit (Bio-Rad, USA). PCR reactions were prepared using a SsoAdvanced Universal SYBR® Green Supermix (Bio-Rad, USA) with gene-specific primers and performed on the CFX Connect real-time system (Bio-Rad, USA) under the following cycling conditions: 95°C for 30 s and 39 cycles for 10 s at 95°C and 30 s at 60°C. An additional step of heating from 65 to 95°C was added to generate a melting curve. Relative mRNA level was calculated using the 2^−ΔΔ^CT method, and Gapdh was used as an internal reference gene.

## 3. Results

### 3.1. Chemical Quality Control of ELAE

**Figure 1:**
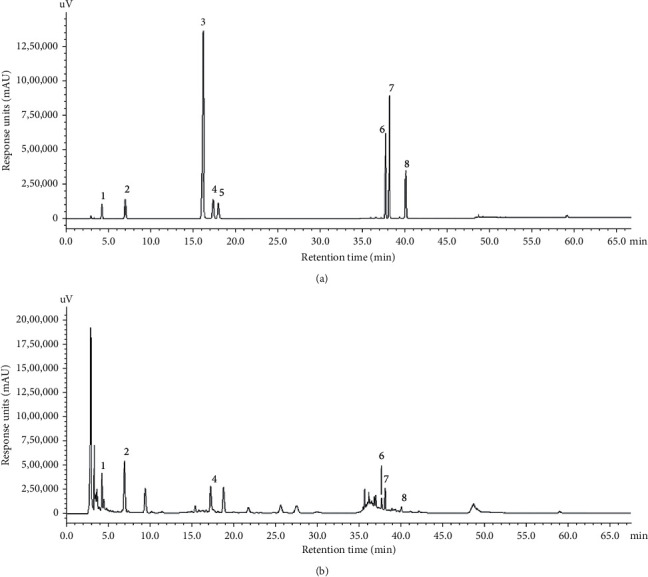
High-performance liquid chromatography (HPLC) of standard chemicals (a) and ELAE extract (b). Eight major components (1 to 8) were identified: 1: aucubin, 2: geniposidic acid, 3: catechin, 4: rutin, 5: caryophyllin, 6: isoquercitrin, 7: chlorogenic acid, and 8: astragaloside. The ordinate represents the signal response units (mAU), while the abscissa represents the retention time (min).

The chemical quality control results of ELAE are shown in Figure 1 and Table 1. Chemical compounds, except carnosine and isoquercitrin, showed the same components as those previously reported, including aucubin, geniposidic acid, rutin, loganin, chlorogenic acid, and astragaloside.

### 3.2. Effect of ELAE on MC3T3-E1 Proliferation

**Figure 2:**
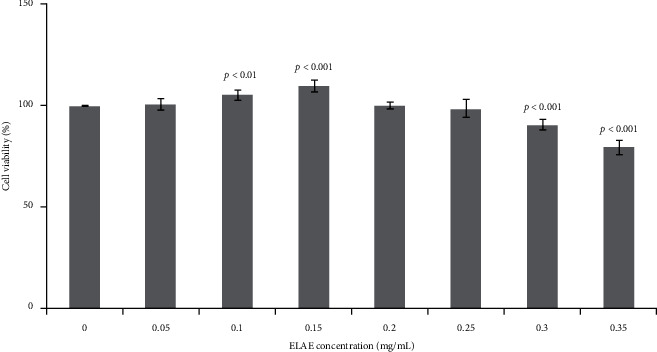
Effects of ELAE on MC3T3-E1 cell proliferation. Cell viability was detected by CCK-8 assay after the treatment with ELAE at progressively increasing concentrations of 0, 0.05, 0.1, 0.15, 0.2, 0.25, and 0.3 mg/mL. Cell viability of the ELAE-treated groups (0.05, 0.1, 0.15, 0.2, 0.25, and 0.3 mg/mL) was estimated by normalizing to cell viability of the untreated group (0 mg/mL). Data are presented as the mean with standard deviation for technical triplicates in an experiment representative of several independent ones. *p* < 0.01 and *p* < 0.001 represent the differences of cell viabilities under ELAE treatment (Student's *t*-test).

The CCK-8 method was used to detect the effect of the aqueous extract of Eucommia leaves on the proliferation of MC3T3-E1 cells. The plain medium was used as a control (Blank group). As shown in Figure 2, the survival rate of MC3T3-E1 osteoblasts treated with ELAE (ELAE group) was significantly increased compared with the untreated control group (0 mg/mL) in a dose-dependent manner, and the effect on cell survival peaked at 0.15 mg/mL. Thus, the concentration of 0.15 mg/mL was chosen for subsequent experiments.

### 3.3. Effect of ELAE on Differentiation and Mineralization of MC3T3-E1 Cells

**Figure 3:**
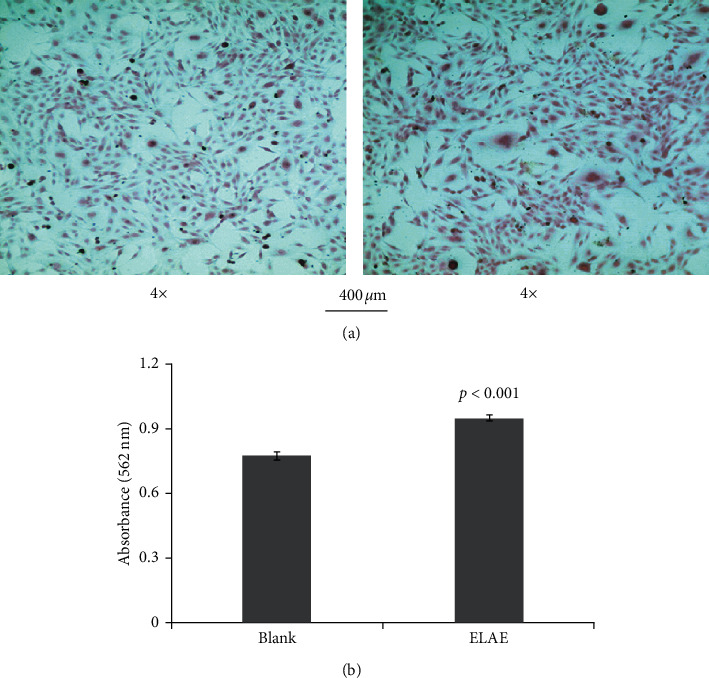
Effect of ELAE on proliferation and differentiation of MC3T3-E1 cells. (a) The deposition of the extracellular matrix of MC3T3-E1 cells was visualized by Alizarin red S staining. (b) The amount of Alizarin red staining was quantified by detecting absorbance at 562 nm.

**Figure 4:**
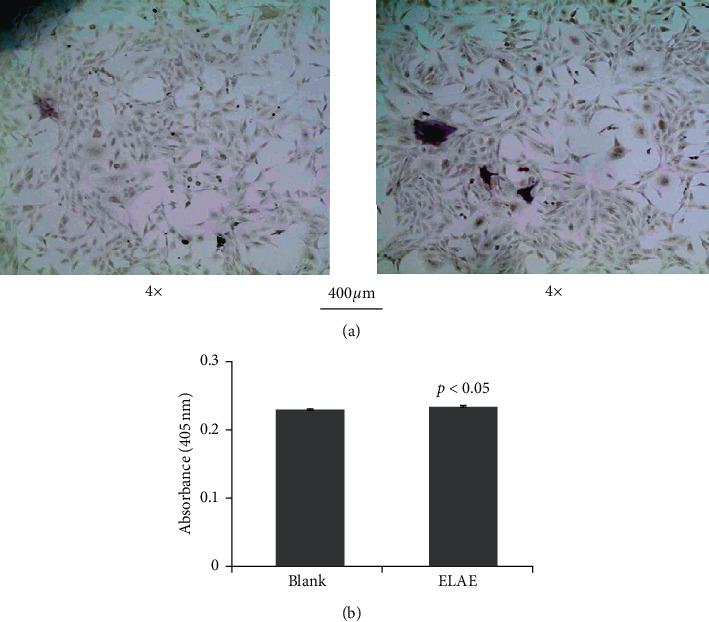
Effect of ELAE on proliferation and differentiation of MC3T3-E1 cells. (a) The deposition of the extracellular matrix of MC3T3-E1 cells was visualized by alkaline phosphatase. (b) The amount of alkaline phosphatase staining was quantified by detecting absorbance at 405 nm.

To evaluate whether ELAE affects the differentiation and maturation of osteoblasts, we examined alkaline phosphatase (ALP) activity and performed Alizarin red S staining. There was a significant increase in ALP and Alizarin red activity compared with the control group (Figures 3 and 4).

### 3.4. Sequencing, Genome Mapping, and Transcript Annotation

As shown in Table 2, after Illumina sequencing and data processing, 41,725,632 and 39,110,394 clean reads were obtained from MC3T3-E1 cells treated with plain medium (Blank group) and those treated with ELAE (ELAE group), respectively. The quality assessment showed that the percentage of *Q*30 was over 93%, and the percentage of GC content was about 52%. For Blank and ELAE-treated samples, 39,579,052 and 37,268,736 clean reads were mapped to the mouse genome, respectively. In total, 13,395 out of 13,512 (Blank group) and 13,471 out of 13,599 (ELAE group) transcripts were annotated by searching against the nonredundant (NR) NCBI protein database and Swiss-Prot database, respectively.

### 3.5. Analysis of Differentially Expressed Genes

We identified 634 genes that were significantly differentially expressed between the ELAE-treated and Blank group (log_2_ fold change ≥1 or ≤−1 and *p* ≤ 0.001), including 30 upregulated genes and 641 downregulated genes in the ELAE group compared with the Blank group, as shown in Table 3.

### 3.6. Functional Enrichment Analysis of DEGs

**Figure 5:**
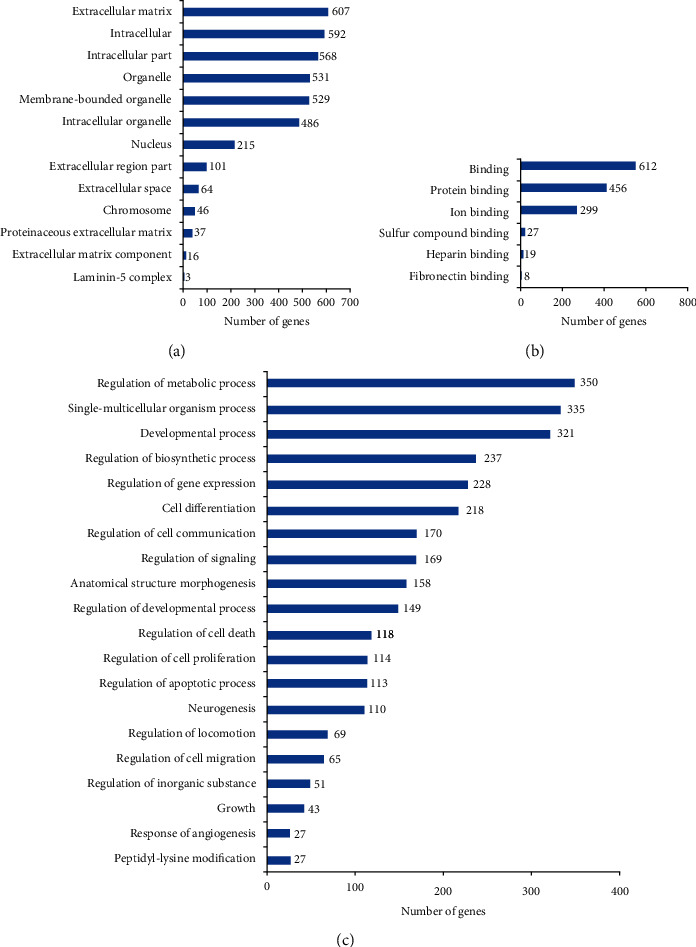
Histogram display of GO enrichment analysis of DEGs. The results are divided into three categories: cellular components, molecular functions, and biological processes. The *x*-axis represents the number of DEGs corresponding to the GO term, and the *y*-axis represents the name of the GO term [15].

According to the functional enrichment analysis of the DEGs, the identified DEGs were divided into the following GO classes: cellular components, molecular functions, and biological process, as shown in Figure 5. The classification of cell components showed that most DEGs are located in the area of extracellular matrix, intracellular part, and organelle. The molecular function classification showed that the main functions of these DEGs are related to the binding activity, such as protein binding and ion binding. The classification of biological processes showed that these DEGs are mainly involved in biological processes, including regulation of the metabolic process, single-multicellular organism process, and developmental process.

### 3.7. Pathway Enrichment Analysis of DEGs

**Figure 6:**
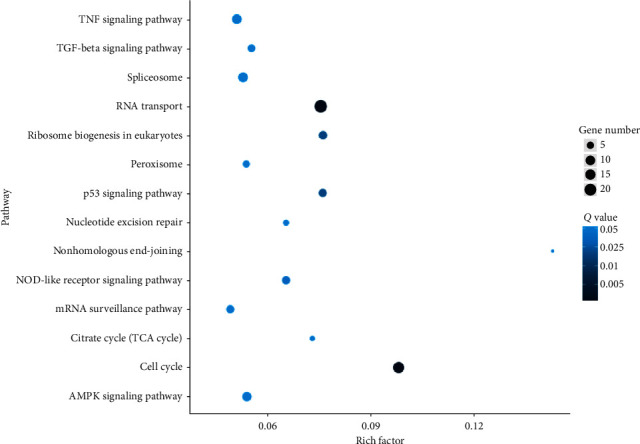
Scatter plot display of KEGG enrichment analysis of DEGs. The *x*-axis represents the enrichment factor, representing the proportion of DEGs involved in the KEGG pathway among all identified DEGs, and the *y*-axis represents the enrichment pathway. The size of the dot reflects the number of DEGs, and the color of the dot reflects the adjusted *p* value (*Q* value).

We further searched the identified DEGs against the KEGG database. The DEGs were mainly mapped to the following signaling pathways: TNF signaling pathway, TGF-beta signaling pathway, spliceosome, RNA transport, ribosome biogenesis in eukaryotes, peroxisome, p53 signaling pathway, nucleotide excision repair, nonhomologous end-joining, NOD-like receptor signaling pathway, mRNA surveillance pathway, citrate cycle (TCA cycle), cell cycle, and AMPK signaling pathway, as shown in Figure 6.

### 3.8. Effects of ELAE on the Expression Levels of Genes Positively Regulating the Proliferation of Cells

Among the identified DEGs, the following DEGs that positively regulate the proliferation of osteoblasts were upregulated: ribosomal protein L10A (Rpl10a), activity-dependent neuroprotective protein (Adnp), peroxisomal biogenesis factor 1 (Pex1), inositol polyphosphate-4-phosphatase type I (Inpp4a), frequently rearranged in advanced T cell lymphomas 2 (Frat2), and protocadherin gamma subfamily A, 1 (Pcdhga1), as shown in Table 4.

### 3.9. Effects of ELAE on the Expression Levels of Genes Negatively Regulating the Proliferation of Cells

Consistent with the above results, the DEGs negatively regulating the proliferation of osteoblasts were downregulated and included secreted nucleophosmin 1 (Npm1), eukaryotic translation initiation factor 3, subunit E (Eif3e), chromobox 3 (Cbx3), proteasome (prosome, macropain) 26S subunit, ATPase, 6 (Psmc6), fibroblast growth factor 7 (Fgf7), fragile *X* mental retardation gene 1, autosomal homolog (Fxr1), DEAD/H (Asp-Glu-Ala-Asp/His) box polypeptide 3, X-linked (Ddx3x), muscle blind-like 1 (Drosophila) (Mbnl1), and cell division cycle 27 (Cdc27), as shown in Table 5.

### 3.10. Effects of ELAE on the Expression Levels of Osteoblast Markers

We further analyzed the expression levels of osteoblast markers under ELAE treatment. The results were consistent with the above findings where ELAE mildly increased the expression levels of multiple osteoblast marker genes, including collagen type5 alpha2 (Col5a2), ubiquitin-associated protein 2-like (Ubap2l), dickkopf WNT signaling pathway inhibitor 3 (Dkk3), forkhead box M1 (Foxm1), collagen type XVI alpha 1 (Col16a1), collagen type XII alpha 1 (Col12a1), ubiquitin-specific peptidase 7 (Usp7), collagen type IV alpha 6 (Col4a6), runt-related transcription factor 2 (Runx2), SRY (sex-determining region Y)-box 4 (Sox4), and bone morphogenetic protein 4 (Bmp4), as shown in Table 6.

### 3.11. qRT-PCR Analysis

**Figure 7:**
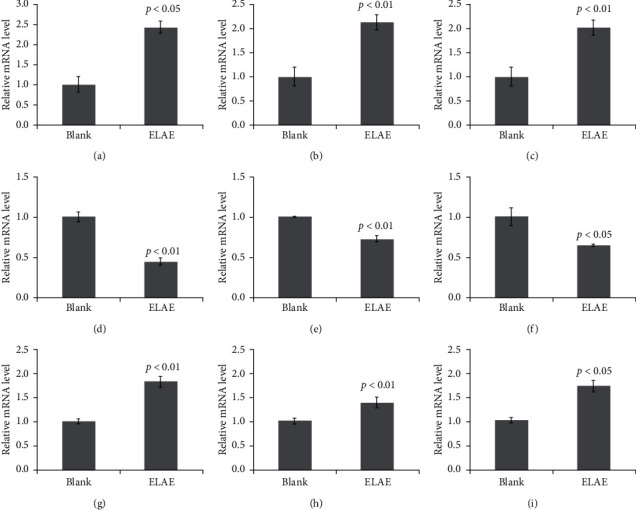
Verification of RNA-seq data by qRT-PCR assay. The relative mRNA levels of the selected DEGs were quantified by qRT-PCR. Data are presented as the mean with standard deviation for technical triplicates in an experiment representative of several independent ones. *p* < 0.05 and *p* < 0.01 represent the differences of relative mRNA levels under ELAE treatment (Student's *t*-test). (a) Rpl10a. (b) Adnp. (c) Pexl. (d) Npm1. (e) Elf3e. (f) Cbx3. (g) Col5a2. (h) Ubap21. (i) Dkk3.

We used qRT-PCR analysis to verify the accuracy of the RNA-seq results. The gene-specific primer sequences including Rpl10a, Adnp, Pex1, Npm1, Eif3e, Cbx3, Col5a2, Ubap2l, Dkk3, and Gapdh are shown in Table 7. The relative mRNA expression levels are shown in Figure 7. The results showed that the results obtained by the qRT-PCR assay were consistent with those obtained by RNA-seq analysis.

## 4. Discussion

Eucommia leaves taste sweet, slightly pungent, and mild in nature. In Shennong's Materia Medica, they are said to be effective for treating waist and knee pain, tonifying the middle, tonifying essence, and strengthening bones and muscles. In modern pharmacology, in vivo and in vitro effects of Eucommia leaves against hypertension, osteoporosis, hyperglycemia, obesity, Alzheimer's disease, aging, diabetes, and sexual dysfunction have attracted much attention [16–21]. It was previously found via traditional Chinese medicine that the active ingredients, including flavonoids, lignans, and iridoid glycosides, can treat osteoporosis and other bone diseases [22]. However, the exact regulatory mechanism remains to be clarified. In the present study, we identified the major components of ELAE which were flavonoids and iridoids, such as aucubin, geniposidic acid, rutin, loganin, chlorogenic acid, and astragaloside. We demonstrated that the survival rate of MC3T3-E1 osteoblasts treated with ELAE was significantly increased compared with the untreated control group in a dose-dependent manner, which is consistent with previously published results [23]. ALP and Alizarin red staining assays further indicated that ELAE significantly increased matrix synthesis and mineralization of MC3T3-E1 cells. We also explored the effects of ELAE on gene expression in MC3T3-E1 cells using RNA-seq technology and qRT-PCR verification method, revealing that ELAE promoted the proliferation of MC3T3-E1 cells and regulated multiple functional genes. These findings suggest that ELAE might become an alternative for the prevention and treatment of bone diseases.

Among the identified genes positively regulating the proliferation of cells, including Rpl10a, Adnp, Pex1, Inpp4a, Frat2, and Pcdhga1, Rpl10a is a component of the 60s subunit of the ribosome. Previous studies indicated that Rpl10a coordinates gene expression during differentiation of preosteoblasts and that the proliferation and differentiation of MC3T3-E1 are accompanied by an increase in Rpl10a [24]. Recent studies suggested that complete deficiency of Adnp in mice results in the inability to form the brain. Adnp also regulates a family of genes associated with bone formation and thus affects the occurrence of osteoblasts [25, 26]. Studies have shown that increased Pex1 expression corresponds to a developmental upregulation of alkaline phosphatase activity in MC3T3-E1 osteoblasts, and Pex1 modulates extracellular matrix turnover through regulation of MMP-9 expression [27, 28]. Inpp4a acts on intracellular calcium levels and can act as a negative regulator of osteoclast differentiation [29]. Studies have shown that Wnt signaling directly reprograms cellular metabolism by stimulating aerobic glycolysis, glutamine catabolism, and fatty acid oxidation in osteoblast-lineage cells [30]. Frat2 and Pcdhga1 affect osteoblasts by influencing Wnt signaling and promoting the proliferation of osteoblasts [31, 32]. Thus, these findings suggest that ELAE regulates osteoblast proliferation and differentiation, thereby promoting bone formation.

Among the identified genes negatively regulating cell proliferation, including Npm1, Eif3e, Cbx3, Psmc6, Fgf7, Fxr1, Ddx3x, Mbnl1, and Cdc27, Npm1, a gene of nuclear phosphoprotein, is involved in the regulation of cell death by mutation, deregulation, or chromosomal translocation in many tumor types, and its reduced expression can promote the proliferation of osteoblasts and the ablation of osteoclasts [33]. Eif3e is an angiogenesis inhibitor and involved in RNA transport, and it is highly expressed in osteoporosis and affects osteoblast apoptosis [34]. Cbx3, a core component of the heterochromatin proteins 1, can inhibit osteoblast differentiation, whereas knocking down its expression can increase the expression of bone-related markers [33]. Psmc6 is a protein that is highly expressed in bone, and Psmc6 gene knockout increases bone mineral density (BMD) and PI3K protein phosphorylation in OVX osteoporosis mice and increases the protein level of cleaved caspase-3/-9 [35]. Ddx3x is an ATPase/RNA helicase of the DEAD-box family, and the increased expression of Ddx3x affects the cell cycle and aggravates apoptosis [36]. Mbn11 is a regulator of RNA metabolism, and its downregulation may promote osteoblast proliferation and inhibit cell apoptosis [37]. Studies have found that Cdc27 can promote cell proliferation and bone growth as well as healing through downregulation, and it has a reverse regulatory effect on osteoblasts [38]. Taken together, these findings suggest that ELAE prevents osteoblast apoptosis and maintains osteoblast homeostasis.

We further showed that ELAE mildly increased the expression levels of osteoblast markers, including Col5a2, Ubap2l, Dkk3, Foxm1, Col16a1, Col12a1, Usp7, Col4a6, Runx2, Sox4, and Bmp4. The effect of collagen molecules on osteoblasts has been confirmed by numerous studies [39]. In this study, we found that Col5a2, Col16a1, Col12a1, and Col4a6 were increased to a different degree. Ubap2l is an important member of the ubiquitin-like protein family. The posttranslational modification of cell proteins with ubiquitin or ubiquitin-like proteins can regulate many cellular processes, such as cell proliferation, differentiation, apoptosis, and signal transduction [40], including in MC3T3-cells [41]. The Wnt/*β*-catenin signaling pathway is crucial in bone development and homeostasis [42]. Overexpression of Foxm1 can partially activate Wnt/*β*-catenin signaling, thereby improving the differentiation of mesenchymal stem cells and promoting the proliferation and differentiation of osteoblasts [43]. Ubiquitin-specific protease 7 (Usp7), also known as herpes virus-associated ubiquitin-specific protease (HAUSP), is one of the most widely studied DUBs. Usp7 can target different proteins to regulate a series of biological processes. For example, it can play an important role in the osteogenic differentiation of human osteoblasts through its catalytic activity [44]. Runx2 is an essential transcription factor for skeletal development and is expressed in multipotent mesenchymal cells, osteoblast-lineage cells, and chondrocytes; it is the main marker in osteogenesis [45]. The SOX protein family plays an important role in bone formation as well. Sox4 is highly expressed in osteoblast progenitor cells, and the Sox4 knockout in osteoblasts reduces the proliferation of progenitor cells and delays the differentiation of osteoblasts [46]. Bmp4, a member of the bone morphogenetic protein family, is an important stimulating factor for osteoblast proliferation and differentiation, bone formation, and stability [47]. Taken together, these findings further support the positive regulatory role of ELAE in promoting osteoblast proliferation, differentiation, and mineralization, as well as in bone formation and repair.

## 5. Conclusions

In the present study, we prepared an aqueous extract of Eucommia leaves (ELAE) and identified its major components including aucubin, geniposidic acid, rutin, loganin, chlorogenic acid, and astragaloside. By conducting RNA-seq analysis combined with proliferation assay, matrix staining detection and verification of gene expression level, and the molecular mechanism of ELAE in the regulation of the osteoblast-like cell line, MC3T3-E1 was explored and deciphered. Based on our findings, ELAE has the potential to promote the proliferation of osteoblasts and consolidate bone formation and stability and has a certain preventive effect on diseases caused by bone dysfunction such as osteoporosis. These findings increase the current understanding of ELAE in regulating bone development and provide theoretical support for the discovery of alternative prevention and treatment options for bone-related diseases.

## Figures and Tables

**Table 1:** Concentration and percentage content of each component in ELAE.

Component	Concentration (mg/mL)	Content (%)
Aucubin	1.3000	3.25
Geniposide	0.7442	1.86
Rutin	0.0519	0.13
Isoquercitrin	0.4515	1.13
Chlorogenic acid	0.3847	0.96
Astragaloside	0.0757	0.38

**Table 2:** Statistical overview of sequencing and mapping results.

Statistics	Blank group	ELAE group
Clean reads	41,725,632	39,110,394
Q30 percentage	93.42	93.79
GC percentage	52.55	52.61
Total mapped reads	39,579,052	37,268,736
Total transcripts	13,512	13,599
Known transcripts	13,395	13,471

**Table 3:** Statistical analysis of DEGs (ELAE vs. Blank).

Statistics	Number
Differentially expressed genes (DEGs)	634
Up regulated genes	30
Down regulated genes	604

**Table 4:** List of DEGs positively regulating the proliferation of osteoblasts (ELAE vs. Blank).

Gene name	Blank (FPKM)	ELAE (FPKM)	log_2_ fold change (ELAE/Blank)	*p* value
Ribosomal protein L10A (Rpl10a)	27.73	63.28	1.19	2.20*E* − 23
Activity-dependent neuroprotective protein (Adnp)	1.91	4.63	1.27	3.76*E* − 13
Peroxisomal biogenesis factor 1 (Pex1)	1.31	2.92	1.15	4.15*E* − 07
Inositol polyphosphate-4-phosphatase, type I (Inpp4a)	1.24	2.87	1.21	1.58*E* − 06
Frequently rearranged in advanced T cell lymphomas 2 (Frat2)	0.05	0.81	4.02	1.79*E* − 04
Protocadherin gamma subfamily A, 1 (Pcdhga1)	0.12	0.64	2.42	3.16*E* − 05

**Table 5:** List of DEGs negatively regulating the proliferation of osteoblasts (ELAE vs. Blank).

Gene name	Blank (FPKM)	ELAE (FPKM)	log_2_ fold change (ELAE/Blank)	*p* value
Nucleophosmin 1 (Npm1)	573.81	191.57	−1.58	0
Eukaryotic translation initiation factor 3, subunit E (Eif3e)	160.26	76.59	−1.07	4.43*E* − 85
Chromobox 3 (Cbx3)	86.54	42.39	−1.03	1.25*E* − 53
Proteasome (prosome, macropain) 26S subunit, ATPase, 6 (Psmc6)	47.95	14.09	−1.77	6.88*E* − 57
Fibroblast growth factor 7 (Fgf7)	44.90	20.09	−1.16	1.73*E* − 50
Fragile *X* mental retardation gene 1, autosomal homolog (Fxr1)	33.40	10.21	−1.71	1.17*E* − 52
DEAD/H (Asp-Glu-Ala-Asp/His) box polypeptide 3, X-linked (Ddx3x)	33.06	8.20	−2.01	3.67*E* − 143
Muscleblind-like 1 (Drosophila) (Mbnl1)	32.34	12.31	−1.40	9.26*E* − 86
Cell division cycle 27 (Cdc27)	8.92	2.33	−1.94	1.95*E* − 48

**Table 6:** Gene list of osteoblast markers (ELAE vs. Blank).

Gene name	Blank (FPKM)	ELAE (FPKM)	log_2_ fold change (ELAE/Blank)	*p* value
Collagen, type V, alpha 2 (Col5a2)	126.18	153.83	0.29	2.32*E* − 38
Ubiquitin-associated protein 2-like (Ubap2l)	68.00	80.93	0.25	3.22*E* − 10
Dickkopf WNT signaling pathway inhibitor 3 (Dkk3)	62.75	75.49	0.27	2.94*E* − 10
Forkhead box M1 (Foxm1)	48.45	55.23	0.19	1.70*E* − 05
Collagen, type XVI, alpha 1 (Col16a1)	46.76	51.28	0.13	1.37*E* − 03
Collagen, type XII, alpha 1 (Col12a1)	20.85	25.79	0.31	4.63*E* − 14
Ubiquitin-specific peptidase 7 (Usp7)	16.46	22.8	0.47	8.63*E* − 13
Collagen, type IV, alpha 6 (Col4a6)	12.25	16.42	0.42	1.02*E* − 09
Runt-related transcription factor 2 (Runx2)	10.05	11.43	0.19	9.47*E* − 03
SRY (sex-determining region Y)-box 4 (Sox4)	3.91	6.85	0.81	3.14*E* − 06
Bone morphogenetic protein 4 (Bmp4)	3.40	5.17	0.60	8.98*E* − 03

**Table 7:** List of qRT-PCR primers for DEGs.

Gene name	Primer	Sequence
Rpl10a	Forward primer	ATGAGCAGCAAAGTCTCACG
	Reverse primer	GGTCGTAGTTCTTCAGGCTGAT

Adnp	Forward primer	GACTCCCACCACGAATCAGC
	Reverse primer	CCCGTTGAATTTAAGTTGGGCT

Pex1	Forward primer	AAGGAAGAGCGTATTAAGCTGGA
	Reverse primer	TCGATTTCCGCACTCTGTTCT

Npm1	Forward primer	ATGGAAGACTCGATGGATATGGA
	Reverse primer	ACCGTTCTTAATGACAACTGGTG

Eif3e	Forward primer	CAGCGAACATGGCTCATTCAT
	Reverse primer	ACACCTACTGTCTGACTGCTTT

Cbx3	Forward primer	ACTGGACCGTCGTGTAGTGAA
	Reverse primer	GCCCCTTGGTTTGTCAGCA

Col5a2	Forward primer	TTGGAAACCTTCTCCATGTCAGA
	Reverse primer	TCCCCAGTGGGTGTTATAGGA

Ubap2l	Forward primer	CAGTCACCTCCAGTAACACGG
	Reverse primer	CGCTGGCAACAGAGAATCA

Dkk3	Forward primer	CTCGGGGGTATTTTGCTGTGT
	Reverse primer	TCCTCCTGAGGGTAGTTGAGA

Gapdh	Forward primer	GCACAGTCAAGGCCGAGAAT
	Reverse primer	GCCTTCTCCATGGTGGTGAA

## Data Availability

The datasets used and/or analyzed during the current study are available from the corresponding author on reasonable request.

## Supplementary Materials

Supplementary Materials Figure S1: the picture of the plant and the leaves of *Eucommia ulmoides*.
