# Supplementary figures and images for: The Aqueous Extract of Eucommia Leaves Promotes Proliferation, Differentiation, and Mineralization of Osteoblast-Like MC3T3-E1 Cells
Source: Evid Based Complement Alternat Med. 2021 Jun 19;2021:3641317. doi: 10.1155/2021/3641317 (PMC8238580; doi:10.1155/2021/3641317)

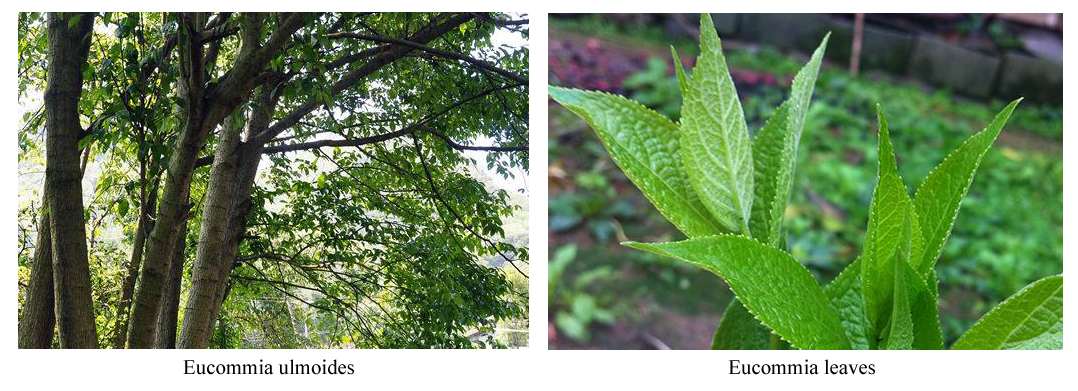


Figure S1: The picture of the plant and the leaves of *Eucommia ulmoides*

Supplement: Supplementary Materials — Figure S1: the picture of the plant and the leaves of Eucommia ulmoides. [file 3641317.f1.docx]
